# Supplementary material for: Trends and Motivations in Dietary Supplement Use Among People with Diabetes: A Population-Based Analysis Using National Health and Nutrition Examination Survey Data from the 2009–2020 Period
Source: Nutrients. 2024 Nov 24;16(23):4021. doi: 10.3390/nu16234021 (PMC11643785; doi:10.3390/nu16234021)
Supplement: Supplementary file 1 [file nutrients-16-04021-s001.zip › Supplementary Materials.pdf]

Supplementary Materials

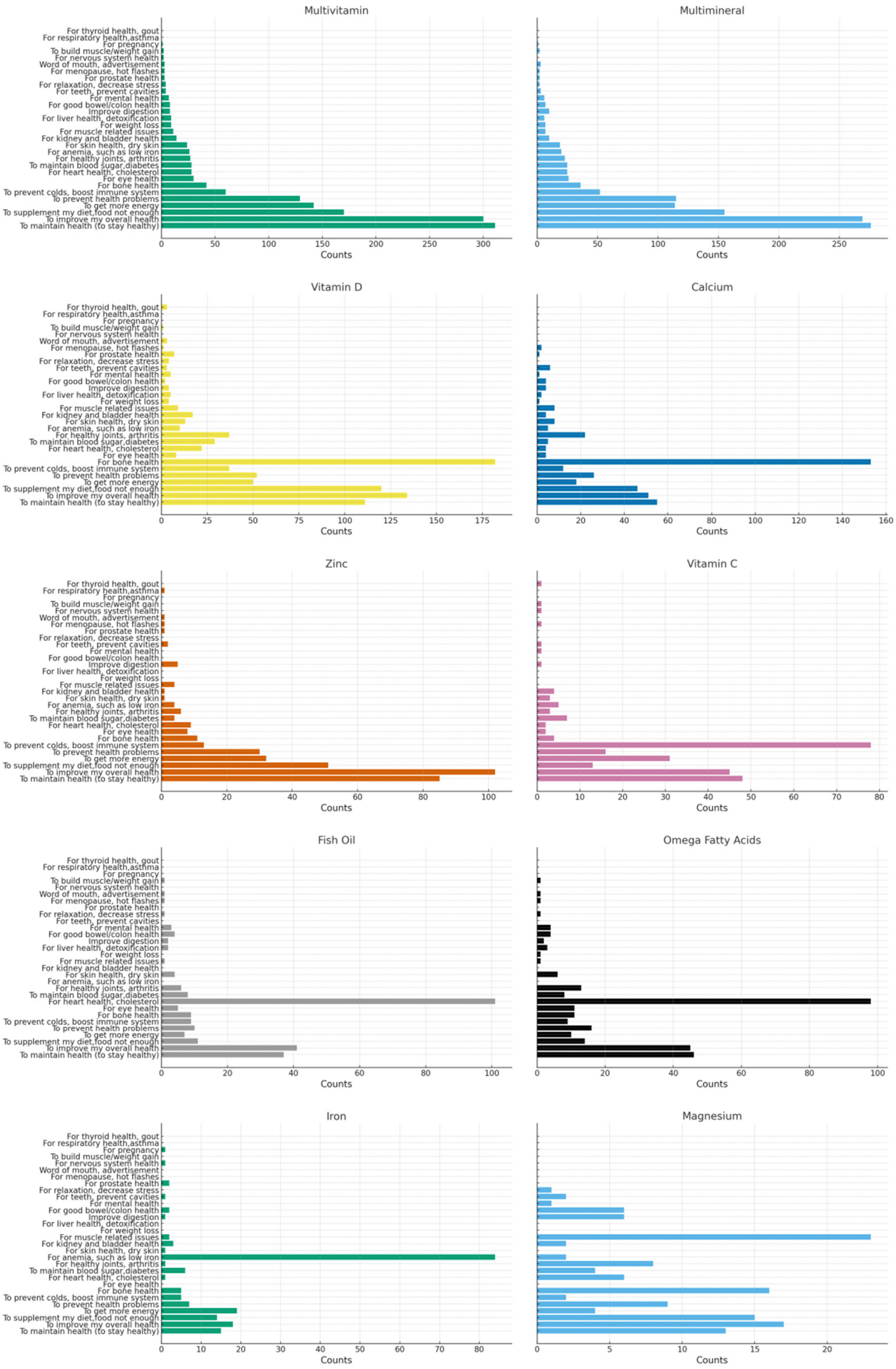

Figure S1. Reasons for using the dietary supplements (top 10 commonly used supplements).

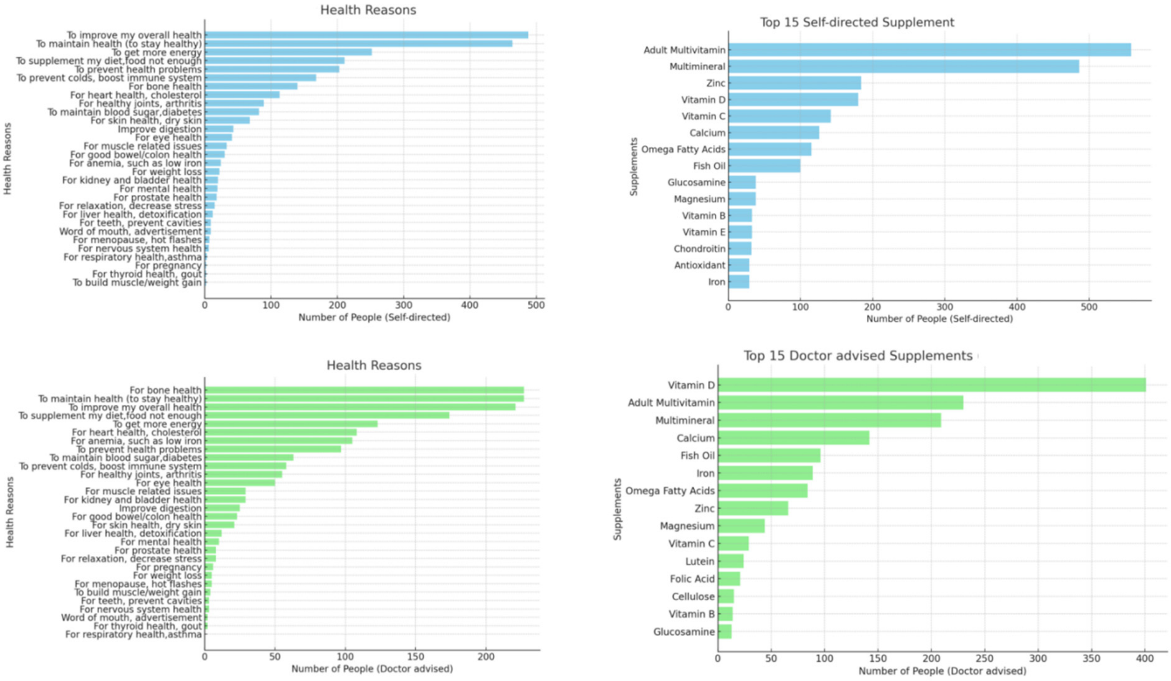

Figure S2. Most commonly used types and reasons for dietary supplement use under doctor-advised and self-directed.
